# Genetic Diversity and Population Structure of Fat-Tailed Coarse-Wooled Sheep Breeds *Ovis aries* from Kazakhstan

**DOI:** 10.3390/vetsci12100988

**Published:** 2025-10-13

**Authors:** Kairat Dossybayev, Daniya Ualiyeva, Tilek Kapassuly, Makpal Amandykova, Altynay Kozhahmet, Bakytzhan Bekmanov, Rauan Amzeyev, Saitou Naruya

**Affiliations:** 1 Laboratory of Animal Genetics and Cytogenetics, RSE Institute of Genetics and Physiology, Science Committee, Ministry of Science and Higher Education of the Republic of Kazakhstan, Almaty 050060, Kazakhstan; kairat1987_11@mail.ru (K.D.);; 2 Department of Molecular Biology and Genetics, Faculty of Biology and Biotechnology, Al-Farabi Kazakh National University, Almaty 050040, Kazakhstan; 3 Laboratory of Molecular Genetic Examination, LLP Kazakh Research Institute of Livestock and Fodder Production, Almaty 050035, Kazakhstan; 4 Institute of Zoology, Science Committee, Ministry of Science and Higher Education of the Republic of Kazakhstan, Almaty 050060, Kazakhstan; 5 Chengdu Institute of Biology, Chinese Academy of Sciences, Chengdu 610213, China; 6 Faculty of Natural Sciences and Geography, Abai Kazakh National Pedagogical University, Almaty 050010, Kazakhstan; 7 Division of Population Genetics, National Institute of Genetics, 1111 Yata, Mishima 411-8540, Shizuoka, Japan; saitounr@nig.ac.jp

**Keywords:** domestic sheep, Central Asia, mtDNA, genetic diversity, population dynamics

## Abstract

Sheep play a central role in Kazakhstan’s pastoral economy, yet the maternal genetic composition of its traditional breeds remains poorly characterized. We analyzed partial mitochondrial D-loop sequences (848 bp) from 115 individuals of three fat-tailed coarse-wooled breeds (Edilbay, Kazakh fat-tailed coarse-wooled, and Gissar) to assess genetic diversity, population structure, and phylogenetic relationships. Ninety-eight haplotypes were identified, indicating high haplotype diversity (Hd = 0.996 ± 0.002) and moderate nucleotide diversity (π = 0.02624 ± 0.00048). Haplotypes clustered into haplogroups A (57.4%) and B (42.6%), with Edilbay dominating the star-like cluster of haplogroup A, consistent with recent expansion. AMOVA revealed that most variation (92.03%) occurred within populations, with no significant differentiation among breeds. Phylogenetic analyses placed Edilbay close to the most recent common ancestor of fat-tailed domestic sheep and the wild *Ovis* species, suggesting retention of an ancestral lineage. These findings highlight Kazakhstan as a genetic crossroads in sheep history and underscore the conservation value of its maternal diversity.

## Simple Summary

Sheep are one of the most important farm animals in Kazakhstan, where traditional breeds such as Edilbay, Kazakh fat-tailed coarse-wooled, and Gissar are still widely raised. However, little is known about their genetic background. In this study, we examined maternal DNA from these breeds to better understand their diversity and origins. We found that they carry many different genetic types, showing both rich diversity and shared ancestry. Most of the genetic variation was found within breeds rather than between them, which suggests that these sheep have a long history of mixing and exchange. Interestingly, the Edilbay breed appears to preserve ancient maternal lineages that connect modern sheep to their wild relatives. Our findings highlight Kazakhstan as an important region in sheep history and emphasize the need to conserve the genetic diversity of its traditional breeds.

## 1. Introduction

Domestic sheep (*Ovis aries*) were among the first livestock species domesticated by humans and remain one of the most widely distributed and economically significant farm animals worldwide. Archaeological and genetic evidence indicate that the Asian mouflon (*Ovis orientalis*) is the wild progenitor of modern domestic sheep, having contributed substantially to their gene pool during the domestication process [1]. Domestication began approximately 9000–10,000 years ago in the Fertile Crescent, a region in the Middle East closely associated with the rise of early agriculture [2,3]. From this origin, sheep dispersed across Eurasia, adapting to diverse environments under both natural pressures and human-driven selection.

Mitochondrial DNA (mtDNA), particularly the hypervariable D-loop region, has proven to be a powerful marker for investigating maternal lineages, population structure, and phylogenetic relationships in domestic sheep [4,5]. To date, six major mtDNA haplogroups (A–F) have been identified globally, with haplogroups A and B predominant, and haplogroup C more frequent in Central Asia. These distributions suggest multiple domestication events or extensive gene flow between wild and domestic populations [6,7,8].

Kazakhstan represents an important center of sheep husbandry, with 33 breeds currently recognized by FAO DAD-IS (2024, accessed on 19 October 2024) [9], many of which are fat-tailed and adapted to harsh climatic conditions. Among them, the Edilbay, Gissar, and Kazakh fat-tailed coarse-wooled breeds are critical genetic resources, valued for their ecological plasticity and meat production [10,11]. The Edilbay breed is believed to have originated from a composite of Kazakh fat-tailed and Kalmyk sheep, and was established at the end of the 19th century in the Ural region (currently the city of Oral in Western Kazakhstan) [10]. Today, it is widespread across Kazakhstan and is regarded as a critical genetic resource. Mature rams can reach weights of up to 103 kg, while ewes typically weigh around 75 kg [11]. The Kazakh fat-tailed coarse-wooled breed is another native breed, developed through the national breeding strategy of Kazakh selectionists. It is distinguished by high precocity and remarkable ecological plasticity, which allow yearlong pasturing under diverse environmental conditions [12]. The Gissar breed, the largest domesticated sheep in the world, was originally developed in the Gissar Valley of Tajikistan and later introduced into Kazakhstan, where it is now distributed in the southern region. Primarily raised for meat, it is notable for its remarkable adaptation to semi-arid environments [13].

Previous genetic studies using mtDNA [5,6,14], microsatellites [11], and SNP arrays [15,16] have revealed high haplotype diversity in Kazakh breeds, strong Asiatic maternal signals, and evidence of long-term lineage continuity from the Bronze Age [17]. However, these studies are fragmented: most focus on individual breeds, use limited sample sizes, or lack a comparative phylogeographic framework integrating Kazakh sheep with global haplogroup data.

As a result, the maternal genetic diversity and evolutionary relationships of fat-tailed breeds within Kazakhstan remain insufficiently understood. Although the genetic history of sheep has been widely studied across Europe [18,19], the Middle East [2,20], and East Asia [21], Central Asian populations, particularly Kazakh fat-tailed coarse-wooled breeds, have been comparatively underrepresented, leaving their role in global domestication and migration processes unclear. Our study advances previous Kazakh sheep research on mtDNA by analyzing a comparatively long 848 bp D-loop fragment across multiple populations, expanding sampling to three fat-tailed coarse-wooled breeds, and embedding the results within a broader Central Asian and Eurasian comparative framework. By integrating local sampling with global haplogroup references and additional GenBank sequences, this work provides the most detailed assessment to date of maternal diversity in Kazakh fat-tailed coarse-wooled sheep and underscores their broader evolutionary and conservation significance, as well as addressing the persistent research gap.

## 2. Materials and Methods

### 2.1. Sampling Information

**Figure 1:**
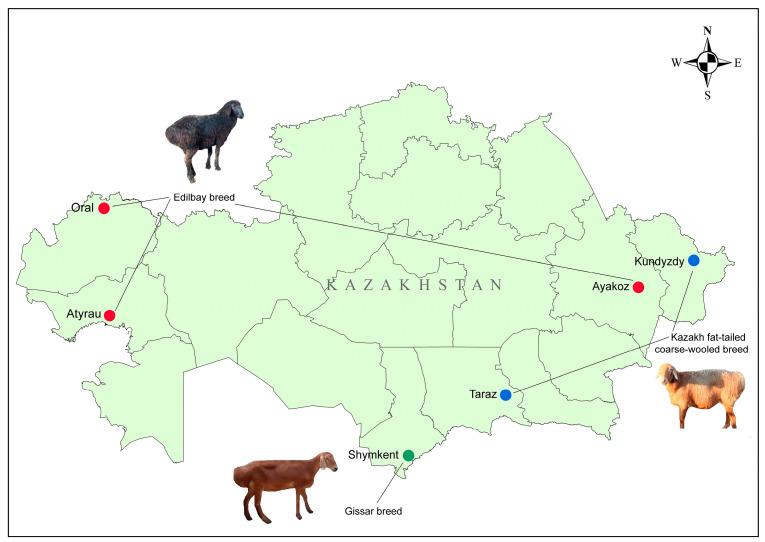
Distribution map of fat-tailed coarse-wooled sheep breeds in Kazakhstan. Photos of each breed representative are from this study, taken by Kapas T. Edilbay breed flocks shown in red color, Gissar breed in green, and Kazakh fat-tailed coarse-wooled breed in blue color.

A total of 115 peripheral blood samples were collected from Kazakhstani *Ovis aries*, represented by three fat-tailed coarse-wool sheep breeds: Gissar (GISS), Kazakh fat-tailed coarse-wool (KFTCW), and Edilbay (ED), with detailed information presented in Table 1 and Figure 1. The Gissar breed is maintained mainly on a single farm in the Turkestan region (South Kazakhstan); therefore, only one flock was employed for sampling. The animals were adult ewes aged 2.5–3 years. Blood collection was performed under the supervision of a veterinary specialist, in accordance with the routine injection program for ruminants, ensuring that no stressful situations were caused. This study was approved by the Local Bioethics Committee of the Institute of Genetics and Physiology of the Republic of Kazakhstan (№12-351 from 23 November 2022). Blood samples were collected using sterile needles and vacuum test tubes containing EDTA (Leuven, Belgium). Samples were transported to the laboratory in portable refrigerated boxes at ±4 °C.

### 2.2. DNA Extraction, Amplification, and Sequencing

Genomic DNA extraction was performed using the GeneJET Genomic DNA Purification Kit (Thermo Scientific Baltics UAB, Vilnius, Lithuania), according to the manufacturer’s protocol. Quality control of the extracted DNA was performed using the NanoDrop One spectrophotometer and Qubit Fluorometer (Thermo Scientific Baltics UAB, Vilnius, Lithuania). The partial sequences of the displacement loop (D-loop) region (849 bp) were amplified using primers F: 5′-AACTCCCAAACATACAACACGG-3′ and R: 5′-ATTTGAGTATTGAGGGCGGGAT-3′ [22]. Polymerase chain reaction (PCR) was carried out in a total volume of 25 μL, containing 0.5–1 μL of genomic DNA with a concentration of 50–100 ng/μL; 1 μL each of forward and reverse primers (concentration of 0.4 μM per primer); 12.5 μL of PCR Master Mix (Thermo Scientific Baltics UAB, Vilnius, Lithuania); and nuclease-free water to complete the volume. The PCR amplification was conducted under the following conditions: pre-denaturation at 95 °C for 10 min, followed by 35 cycles of 95 °C for 45 s, 55–59 °C for 45 s, and 72 °C for 60 s. A final extension was performed at 72 °C for 15 min, and the reaction was terminated at 4 °C. The PCR products were assessed using 1% agarose gel electrophoresis, purified, and sequenced in both directions using the PCR primers on the SeqStudio Genetic Analyzer (Applied Biosystems, Thermo Fisher Scientific, Waltham, MA, USA) at the Laboratory of Animal Genetics and Cytogenetics of the Institute of Genetics and Physiology of the Republic of Kazakhstan. Forward and reverse reads were assembled into consensus sequences using SeqMan (Lasergene package; DNASTAR Inc., Madison, WI, USA) [23]. Chromatograms were visually inspected, and low-quality ends were trimmed prior to alignment. Only high-quality sequences were retained for downstream analyses.

### 2.3. Molecular Diversity and Population Dynamics Analyses

All 115 obtained DNA sequences of the D-loop region (849 bp) were aligned with an *Ovis aries* reference sequence of haplogroup B (NC_001941) [19] using the MAFFT v.7.313 [24] under the default parameters as implemented in PhyloSuite v.1.2.2 [25] and have been deposited in GenBank with the accession numbers PV963845-PV963959 (Table S1). Haplotypes were extracted using DnaSP 5.0 [26], as well as molecular diversity indices for each breed, including the number of haplotypes (h), nucleotide diversity (π), haplotype diversity (Hd), average number of nucleotide differences (k), number of segregating sites (S), and the number of mutations (m). Uncorrected sequence divergence (*p*-distances) was calculated in MEGA v. 11 software [27] between analyzed breeds of Kazakhstani fat-tailed coarse-wooled sheep. To identify which haplogroup the Kazakhstani sheep breeds belong to, we conducted median–joining haplotype network analysis using Network v4.6.0 (Fluxus Technology Ltd., UK) (accessed on 15 December 2024) [28].

Population dynamic analysis was conducted to estimate mismatch distribution and neutrality indices (Fu and Li’s *D* and *F* tests) [29] using DnaSP 5.0. These statistics were selected over other neutrality tests (Tajima’s *D*, Fu’s *Fs*) because they are particularly sensitive to the excess of rare variants on external branches of the genealogy, making them well-suited for detecting recent demographic expansion or purifying selection in mitochondrial DNA sequences. For completeness, we also calculated Tajima’s *D* and Fu’s *Fs*, which are widely reported in mtDNA studies, and provided in Supplementary Table S2.

Mismatch distribution indices, including Harpending’s raggedness index (Rag) and Ramos-Onsins and Rozas’ R^2^ statistic, were calculated in DnaSP v5.0 [26] to summarize demographic patterns. Sum of squared deviations (SSD) and goodness-of-fit *p*-values were not reported as model fitting did not converge for the D-loop data.

Genetic variation within populations, among populations within groups, and between groups was assessed by the analysis of molecular variance (AMOVA) carried out in the program Arlequin v.3.11 [30], with the significance test based on 1000 permutations. To visualize the genetic structure among breeds, Principal Coordinate Analysis (PCoA) was performed in R v.4.4.1 using the *ape* [31] and *vegan* packages. Pairwise genetic distances were calculated from mitochondrial D-loop sequences under the Tamura–Nei (TN93) substitution model, which accounts for unequal base frequencies and transition/transversion rate biases characteristic of mitochondrial DNA. The resulting distance matrix was subjected to PCoA (*cmdscale* function), and the first two principal coordinates were plotted in two-dimensional space.

### 2.4. Phylogenetic Analysis

Phylogenetic analysis was conducted based on 144 sequences of D-loop region, including 115 samples generated in this study, 10 sequences of *Ovis aries* available in GenBank representing five haplogroups (A–E) [6], 9 sequences representing Edilbay and Gissar breeds, as well as 4 sequences of sheep closely related to Kazakhstani indigenous breeds—such as the Kalmyk breed [32]—that were retrieved from GenBank. Additionally, we included wild representatives—*Ovis orientalis* (Asian mouflon) [33] and *O. orientalis ophion* (also known as *O. gmelini ophion*—Cyprus mouflon) [34]—and two outgroups—*Ovis ammon* (argali) [6] and *Ovis vignei* (Urial) [33] (Table S1)—which were chosen due to their close evolutionary relationships within the genus *Ovis*.

Phylogenetic trees were reconstructed using Maximum Likelihood (ML) implemented in IQ-TREE v.1.6.8 [35] under the HKY + G + I model selected as the best-fit model using PartitionFinder in PhyloSuite v.1.2.2 under the Bayesian Information Criterion (BIC). Branch support analyses were conducted under 5000 ultrafast-bootstrap replicates (UFBoot) [36]. Nodes with UFBoot ≥ 90 were considered to be well-supported [36]. The phylogenetic trees were viewed and edited with iTOL v6 (https://itol.embl.de) (accessed on 15 January 2025) [37].

## 3. Results

### 3.1. MtDNA Diversity and Population Structure of Kazakhstani Sheep Breeds

Molecular diversity indices calculated for the partial segment of the mitochondrial D-loop region (848 bp) obtained from 115 Kazakhstani fat-tailed sheep revealed consistently high genetic variation across all three studied breeds. Haplotype diversity (Hd) was high, with values ranging from 0.976 in KFTCW to 0.988 in the ED breed, while the GISS breed exhibited the highest diversity (0.997), indicating a high number of unique haplotypes per breed. Nucleotide diversity (π) ranged from 0.02187 (GISS) to 0.02528 (KFTCW) and 0.02736 (ED), reflecting the more variable nature of the control region (Table 2).

Pairwise uncorrected sequence divergences (*p*-distances) were calculated among the Kazakhstani fat-tailed coarse-wool sheep breeds, which revealed relatively low levels of genetic divergence, indicating overall close maternal relationships among the breeds. The lowest genetic distance was observed between the Edilbay and Gissar breeds (0.0252), followed closely by Gissar and KFTCW (0.0258), and KFTCW and Edilbay (0.0267).

**Figure 2:**
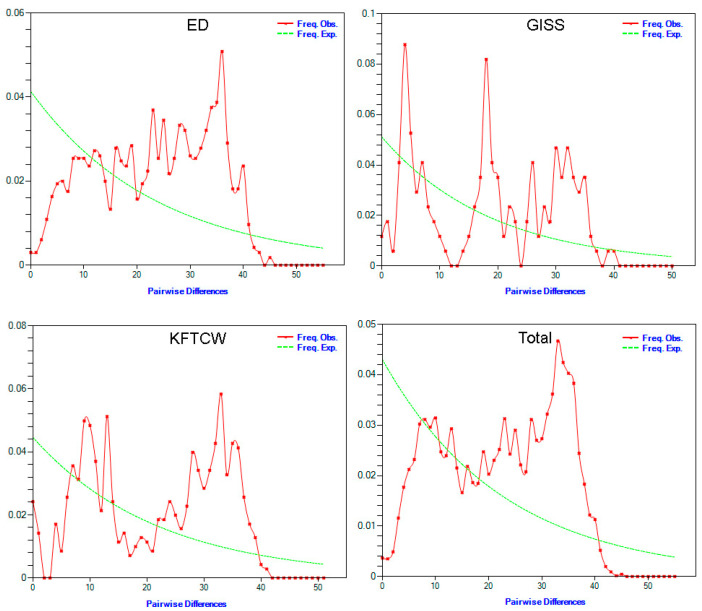
Mismatch distribution analysis of analyzed sheep from Kazakhstan.

Neutrality tests using Fu and Li’s *D* and *F* statistics produced non-significant positive values for all breeds, indicating no strong deviation from neutrality (Table 3). Tajima’s *D* values were likewise non-significant (ED = 0.0079, KFTCW = 0.6404, and GISS = 0.3688), whereas Fu’s *Fs* values were significantly negative (ED = −24.11, KFTCW = −19.66, and GISS = −6.11; all *p* < 0.05), consistent with recent demographic expansion. Mismatch distribution analysis, assessed through the raggedness index (Rag) and R^2^ statistic, provided more insight into demographic patterns. Low Rag and R^2^ values in ED and the total dataset (Rag = 0.0024, R^2^ = 0.1155 for ED; Rag = 0.0012, R^2^ = 0.0927 for total) are compatible with recent demographic expansion under a sudden expansion model (Table 3). In contrast, higher values in KFTCW and GISS suggest either population stability, genetic structure, or a lack of recent expansion (Figure 2). In some cases, the calculation of SSD and *p*-values did not converge in Arlequin, likely reflecting complex demographic histories and moderate sample sizes. Nevertheless, the concordance of Fu’s *Fs* with mismatch-based indices (Rag, R^2^) and observed vs. expected curves supports a demographic expansion signal in Edilbay and in the pooled dataset.

**Figure 3:**
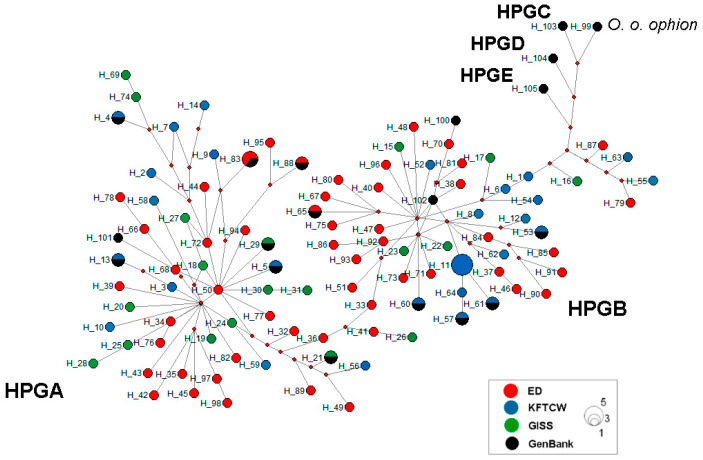
Median-joining haplotype network based on mitochondrial D-loop sequences from Kazakhstani fat-tailed coarse-wooled sheep breeds. Circle size is proportional to haplotype frequency (largest circle = 5 sequences; medium circle = 3 sequences; smallest circle = 1 sequence). Colors represent breed populations as shown in Figure 1. All sequences obtained from GenBank are shown in black. Red small dots represent median vectors.

A median-joining haplotype network was constructed based on D-loop sequences to explore the maternal lineage structure of Kazakhstani fat-tailed coarse-wooled sheep breeds. The haplotype network identified two major haplogroups—HPGA and HPGB—which included the vast majority of haplotypes from the studied populations (Figure 3). Haplogroup A (HPGA) formed a large star-like cluster with short mutational distances between haplotypes, suggesting a recent population expansion. This cluster was dominated by Edilbay (ED)-breed individuals, and also included numerous haplotypes from Kazakh fat-tailed coarse-wooled (KFTCW) and Gissar (GISS) breeds, indicating shared maternal ancestry among these populations. Haplogroup B (HPGB) showed a more dispersed structure, with longer mutational branches separating haplotypes. It included individuals from all three studied breeds, but with stronger representation from KFTCW and GISS, reflecting a degree of maternal lineage divergence and possibly historical admixture. In contrast, haplogroups C (HPGC), D (HPGD), and E (HPGE) were represented exclusively by reference haplotypes and contained no Kazakhstani sheep. This indicates that the studied breeds are not maternally descended from these rarer or regionally restricted haplogroups. Among the 124 haplotypes identified (98 from our dataset), most were unique to single individuals. Several haplotypes were shared by two to three individuals within the same population (e.g., AKZ, ATR, KDZ, SHK, and TRZ), while only one haplotype (H11) was shared between populations (KDZ and TRZ) within the KFTCW breed. No haplotypes were shared across breeds.

To assess the genetic structure among the three fat-tailed coarse-wooled sheep breeds (ED, KFTCW, and GISS), an Analysis of Molecular Variance (AMOVA) was performed using a hierarchical design, where six populations were grouped into three breed-level groups. The AMOVA included all three breeds: ED (comprising the populations ORL, ATR, and AKZ), KFTCW (populations TRZ and KDZ), and GISS (represented solely by population SHK). The results indicated that most of the genetic variation was found within populations (92.03%), while a smaller but significant portion was attributed to differences among populations within breeds (11.03%; FSC = 0.10706, *p* = 0.00196). However, no significant variation was detected among breeds (−3.06%; FCT = −0.03060, *p* = 0.63148). The negative variance component does not imply an actual loss of genetic variation, but rather indicates that differentiation among breeds is negligible, with most genetic diversity residing within populations rather than between groups. The overall genetic differentiation was moderate (FST = 0.07974, *p* = 0.00098) (Table 4). It should be noted that the GISS breed was represented by only one population (SHK), which may have limited the detection of among-group variation, as variance components within that group could not be estimated. Nonetheless, the inclusion of GISS allowed for a complete breed-level comparison.

**Figure 4:**
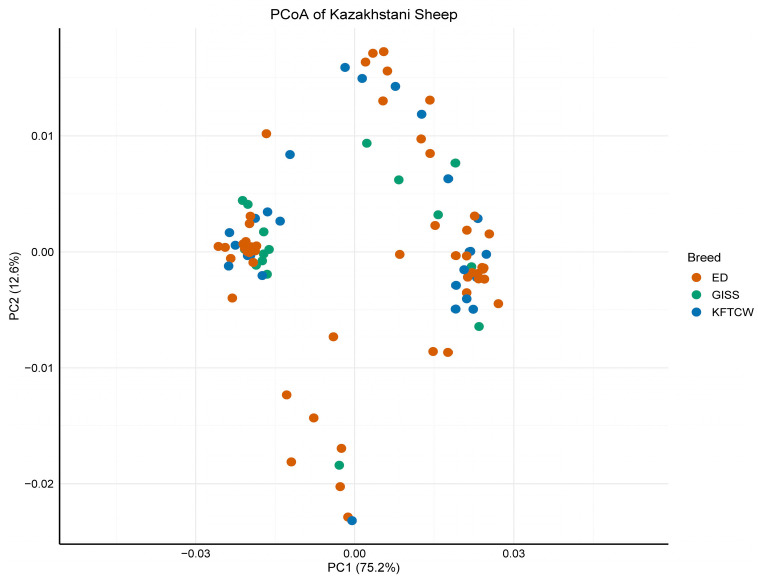
Principal Coordinate Analysis (PCoA) of 115 Kazakhstani sheep individuals based on TN93—pairwise genetic distances. Points represent individual samples, colored by breed according to Figure 1. The first two principal coordinates explain 87.8% of the total variation (75.2% and 12.6%, respectively).

To consider the genetic differentiation among the three Kazakh sheep breeds, PCoA was examined based on pairwise distances of mitochondrial D-loop sequences. The first two axes explained 87.8% of the total variation (75.2% and 12.6%, respectively; Figure 4). Samples clustered largely according to their breeds, with partial overlap between the ED, GISS, and KFTCW groups, indicating shared maternal lineages or historical gene flow. Several samples fell outside their breed’s 95% confidence ellipse, suggesting the presence of rare haplotypes or potential admixture events.

### 3.2. Phylogenetic Relationships

**Figure 5:**
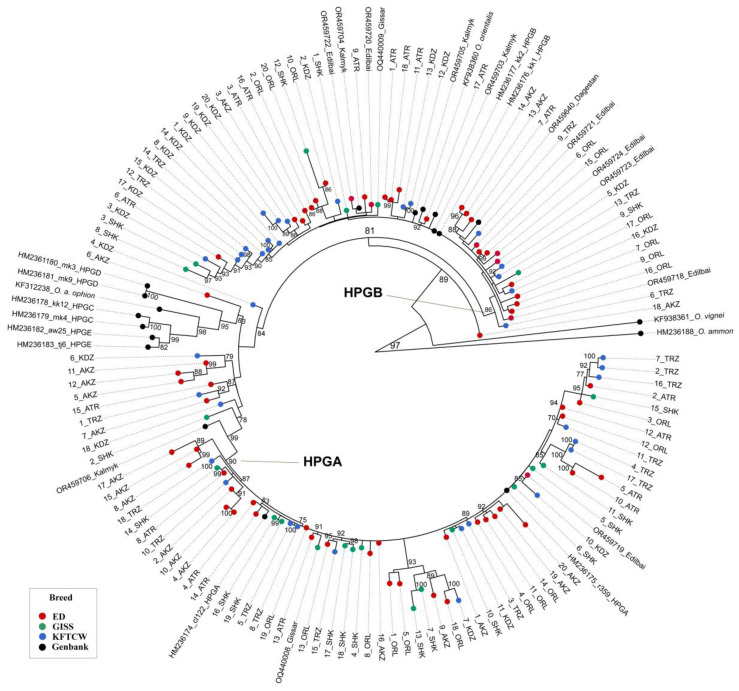
ML tree demonstrating the phylogenetic relationships of Kazakhstani fat-tailed coarse-wooled sheep breeds with wild representatives, and five main haplogroups of *O. aries*.

The Maximum Likelihood (ML) phylogenetic tree reconstructed from 143 mitochondrial D-loop sequences (849 bp) resolved two major domestic sheep clades corresponding to haplogroups A (HPGA) and B (HPGB) (Figure 5). The majority of Kazakhstani sheep sequences clustered within haplogroup A (HPGA), which accounted for 57.4% (57/105) of the analyzed samples, including Edilbay (30), Gissar (13), and Kazakh fat-tailed coarse-wooled (KFTCW) sheep (14). The remaining 42.6% (48/105) of sequences belonged to haplogroup B (HPGB), comprising Edilbay (23), Gissar (5), and KFTCW (20) individuals. Thus, HPGA was the predominant maternal lineage in Kazakhstani sheep, a pattern consistent with the dominance of this haplogroup in other Central Asian sheep populations reported in previous studies [2,18]. Within HPGB, *Ovis orientalis* (Asian mouflon) clustered alongside a subset of Kazakhstani domestic sheep, representing a mixture of Edilbay, Kazakh fat-tailed coarse-wooled, and Gissar breeds, consistent with earlier findings of haplogroup B being shared between domestic sheep and wild Asiatic mouflon [34,38].

Importantly, we identified a distinct intermediate branch that diverged after the wild *Ovis* lineages and the minor haplogroups C, D, and E, but before the main HPGA clade. This branch comprised sequences from multiple Kazakhstani populations, including Edilbay (AKZ n = 5), Gissar (SHK n = 1), and Kazakh fat-tailed coarse-wooled sheep (TRZ n = 1, KDZ n = 1), along with one Kalmyk breed sequence (OR459706). Having a median bootstrap support, this grouping may represent a transitional clade between the more ancient haplogroups (C, D, and E) and the predominant domestic haplogroups A and B, reflecting a lineage that retained ancestral diversity while contributing to the maternal pool of Central Asian sheep. Its composition, integrating several Kazakhstani breeds and a Kalmyk sample, points to a shared evolutionary trajectory shaped by the Eurasian steppe pastoralism corridor. Additionally, a sub-branch of Edilbay-breed individuals (population AKZ) diverged directly after the wild *Ovis* lineages (BS = 89%), reinforcing the possibility of an ancestral position of this breed within Central Asian sheep or a closer relationship to wild ancestors, as previously hypothesized by Hiendleder et al. (1998) [19].

Sequences representing haplogroups C, D, and E formed distinct peripheral clades outside the Kazakhstani clusters, confirming their absence in the analyzed populations. Outgroups (*O. vignei* and *O. ammon*) were placed basal to the domestic sheep clades as expected. Ultrafast-bootstrap support values (UFBoot) were high (≥90) for the nodes defining major haplogroups, supporting the robustness of the higher-level topology.

## 4. Discussion

### 4.1. Maternal Genetic Diversity and Regional Comparisons

Mitochondrial DNA is among the most widely used molecular tools for investigating genetic diversity and inferring the maternal origins of domestic sheep [19,39,40]. Our analysis of partial D-loop sequences (848 bp) from Kazakhstani fat-tailed breeds revealed high haplotype diversity (Hd = 0.996) and moderate nucleotide diversity (π = 0.02624), indicative of substantial maternal variability. This overall pattern is consistent with the general trend for Eurasian sheep but differs in the relative composition of haplogroups. The nearly balanced proportions of HPGA (57.4%) and HPGB (42.6%) are unusual compared to many neighboring populations, where one haplogroup typically dominates. Notably, haplogroups C, D, and E were absent from our dataset.

Comparative data from previous studies help contextualize these findings. In Russian native sheep, Koshkina et al. (2023) [32] reported a predominance of HPGB (64.8%), with HPGA at 28.9%, and minor contributions from HPGC (5.5%) and HPGD (0.8%) based on whole mitochondrial genomes. Similarly, Kalaydzhiev et al. (2023) [41] found very high haplotype diversity in Bulgarian native breeds, yet with an overwhelming frequency of HPGB (95.2%) and only 4.8% HPGA. These contrasts underscore the unique intermediate position of Kazakhstan, where both eastern HPGA-rich and western HPGB-rich lineages appear to have converged and mixed over time.

Comparable evidence from other Central Asian populations further contextualizes these results. In Mongolia, mtDNA D-loop analyses reveal high haplotype diversity (Hd ≈ 0.97), with haplogroup A predominating, followed by B and low-frequency C, while haplogroups D and E are largely absent [42]. This reflects broad maternal variation with limited geographic partitioning, consistent with centuries of nomadic herd mobility. In Kyrgyzstan, although most recent work has employed microsatellites and SNPs rather than mtDNA, local breeds display high within-breed diversity and shallow subdivision, highlighting weak maternal structuring under open pastoral systems [43]. In Uzbekistan, modern mtDNA datasets remain scarce, but ancient mitogenomes dated to ca. 4400–3100 BP document the co-occurrence of multiple maternal lineages, supporting long-term lineage complexity along the steppe corridor [44]. Together, these regional patterns include Mongolia’s A-biased pool, Kyrgyzstan’s weak subdivision, and Uzbekistan’s early multi-lineage signals, which frame Kazakhstan’s balanced A/B composition as part of a broader Central Asian mosaic shaped by sustained pastoral mobility and admixture.

Our findings also align with Mukhametzharova et al. (2018) [5], who analyzed a 539 bp fragment of mtDNA D-loop in six Kazakh sheep breeds. Their study identified three haplogroups, A (43.75%), B (39.06%), and C (17.19%), with haplogroups D and E absent. Notably, the Edilbay breed was represented only within haplogroups A and B. The detection of haplogroup C in their dataset was confined to other breeds (semi-fine-wooled types) that had undergone crossbreeding with European sheep aimed at productivity improvement.

On the other hand, archaeogenetic evidence reinforces the persistence of major haplogroups over time. Tarlykov et al. (2021) [17] examined Bronze Age sheep from Kazakhstan and found haplogroups A (57%), B (36%), and C (7%), proportions remarkably similar to those in modern populations. This suggests long-term continuity of maternal lineages despite centuries of breeding, migration, and transhumance.

The high mitochondrial diversity observed across Kazakhstani fat-tailed coarse-wooled breeds, particularly Edilbay, aligns with the view that Central Asia represents a major center of sheep genetic diversity and post-domestication expansion. However, given that our analysis is based on partial D-loop sequences, such interpretations should be regarded as hypotheses until validated by whole-mitogenome or nuclear genome evidence. This interpretation is consistent with studies highlighting the Iranian Plateau and the Caucasus—both geographically proximate to Kazakhstan—as regions harboring high mtDNA variation due to their roles in early sheep dispersal [45]. The elevated haplotype and nucleotide diversity in the D-loop region reflect both the natural mutability of mitochondrial control sequences and the preservation of multiple maternal lineages within Kazakh populations. Comparable diversity patterns have been reported in Turkish and Iranian native sheep [2,46], where transcontinental trade routes such as the Silk Road likely facilitated long-distance gene flow.

### 4.2. Population Dynamics and Structure

Demographic- and structure-oriented analyses indicate a maternal gene pool characterized by recent growth in parts of the dataset and weak breed-level subdivision overall. The mismatch distribution supports expansion in the Edilbay breed and in the pooled sample (low raggedness and R^2^), whereas KFTCW and GISS show higher indices compatible with relative stability or latent structure. The lack of convergence for SSD and *p*-values likely reflects a complex demographic history and moderate sample sizes rather than data quality issues. Therefore, mismatch results should be considered alongside neutrality tests and phylogenetic evidence to support demographic interpretations. While Fu and Li’s statistics and Tajima’s *D* showed no strong deviation from neutrality, Fu’s *Fs* and mismatch indices indicated expansion in Edilbay and the pooled dataset. This discrepancy likely reflects the greater sensitivity of Fu’s *Fs* and mismatch analyses to recent demographic events, compared with the more conservative nature of Fu and Li’s and Tajima’s statistics under moderate sample sizes.

The star-like haplotype configuration of HPGA, dominated by Edilbay individuals, resembles expansion signals observed in wild-to-domestic transition studies [19], potentially reflecting a founder effect or rapid demographic growth. In contrast, the broader haplotype spread in HPGB and the presence of more divergent lineages in KFTCW and Gissar suggest historical introgression or the retention of distinct maternal origins, particularly plausible for the Gissar breed, which has known external origins. The lack of significant genetic differentiation among breeds in the AMOVA despite strong within-population variation parallels findings from northern Eurasia and the Baltic region, where livestock mobility and breeding practices have blurred phylogeographic boundaries [18]. Collectively, these results highlight a shared maternal ancestry across Kazakhstani sheep breeds, with Edilbay potentially acting as a genetic reservoir, while Gissar and KFTCW reflect more recent admixture and localized evolution.

The AMOVA attributes 92.03% of variance to within-population differences, 11.03% to among-population within-breed differences, and no significant variance among breeds. The slightly negative among-group component (−3.06%) further indicates an absence of meaningful breed-level structure, consistent with the PCoA showing overlapping clusters and high within-flock diversity. These conditions are expected under sustained exchange of ewes and rams across the steppe, where seasonal movements and trade have historically blurred maternal boundaries. It should be noted that the Gissar breed was sampled from a single flock, which limits breed-level generalization; however, the consistency across all three breeds supports the robustness of the observed diversity and structure patterns. This interpretation is further supported by the haplotype distribution. Nearly all haplotypes were unique to single individuals or populations, with only one haplotype (H11) shared between KDZ and TRZ within the KFTCW breed, and none shared across breeds. Such a pattern underscores the predominance of within-population diversity and the limited extent of maternal overlap between breeds.

Comparable signals appear in neighboring regions. Mongolian D-loop studies report high haplotype diversity with little geographic partitioning, attributed to centuries of nomadic herd management and open-range movements [47]. Indian sheep show high within-population variance and modest regional differences based on combined mtDNA and microsatellite data [48]. Recent data from Pakistan (Khyber Pakhtunkhwa; D-loop; 159 individuals) also show very-high haplotype diversity (Hd = 0.985) with three maternal lineages (A, B, and C), pointing to broad admixture within provinces [49]. Similarly, Iranian sheep exhibit high diversity and within-population variance near putative domestication centers [50].

These results fit broader models of post-domestication movements, where repeated dispersals and secondary introductions erode the fine-scale structure unless barriers or strict management enforce isolation [51]. The absence of haplogroups C–E in our dataset does not contradict these dynamics. Rare haplogroups may be missed due to moderate sample sizes or short sequence fragments. Studies using the complete D-loop or whole mitogenomes often detect rare haplotypes overlooked in partial analyses [52].

In summary, the expansion signatures (mismatch), weak breed differentiation (AMOVA and PCoA), and very-low between-breed *p*-distances together point to a historically panmictic maternal system in Kazakhstan. This pattern is consistent with other steppe populations, where demographic growth and sustained inter-flock connectivity, rather than breed isolation, shaped maternal structure.

### 4.3. Phylogenetic Relationships Among Kazakhstani Sheep Breeds

Our phylogenetic reconstruction places Kazakhstani sheep predominantly within haplogroups A and B, but with a distinctive near-equal balance between the two (HPGA = 54.3%; HPGB = 45.7%). This contrasts with the dominance of HPGA in most East and Central Asian flocks [2,19] and the near-fixation of HPGB in several Balkan and Caucasian populations [18,53]. Such a balance is consistent with Kazakhstan’s position as a contact zone where eastern HPGA-rich and western HPGB-rich lineages converged, likely through repeated livestock exchanges along the Eurasian steppe corridor, particularly during the Silk Road period [54].

A notable feature is the placement of Edilbay close to the node linking domestic sheep with *O. vignei* and *O. ammon.* This position suggests that Edilbay could represent a hypothesized relic of an ancestral Central Asian lineage, although this interpretation remains tentative due to reliance on partial mtDNA sequences. Alternative explanations such as incomplete lineage sorting, limited sampling, or stochastic retention of basal haplotypes must also be considered.

The clustering of *O. orientalis* (Asiatic mouflon) within HPGB agrees with earlier genetic evidence for its role as a direct maternal contributor to haplogroup B [1,19]. The relatively high HPGB proportion in our dataset, higher than in some Iranian and Anatolian flocks [7,18], may reflect secondary introgression from western flocks or selective breeding preferences favoring HPGB lineages.

Intermediate clades between minor haplogroups (C, D, and E) and the main domestic clusters of HPGA and B (Figure 3 and Figure 4) suggest that Central Asian flocks—particularly Edilbay (hypothetically)—may serve as a genetic bridge connecting Asian, European, and Middle Eastern lineages. Similar transitional haplotypes have been reported in Anatolian mouflon, Caucasian sheep [7,55], and hybrids such as Iranian Kerman sheep [38]. Their presence across Edilbay, Gissar, and KFTCW indicates that such lineages reflect a shared maternal heritage of steppe pastoralism rather than isolated events.

These findings parallel paleogenomic evidence from Bronze Age sheep in Central Asia, which show mixtures of Near Eastern, Caucasian, and steppe lineages persisting into modern breeds [54]. Thus, while Edilbay may preserve genetic traces of ancient diversity, its precise status requires further study with higher-resolution genomic tools. The combination of Edilbay’s proximity to wild *Ovis* MRCA, the balanced A/B haplogroup composition, and the presence of transitional lineages underscores Kazakhstan’s position as a genetic and cultural crossroads in sheep history.

### 4.4. Implications for Breed Conservation and Genetic Management

The balanced yet diverse maternal lineage pool in Kazakhstani sheep positions them as both a crucial conservation resource and a reservoir for breed improvement. The prevalence of two major haplogroups, combined with potential remnants of rarer lineages, provides adaptive potential not always available in more lineage-homogenous populations like those of the Balkans or South Asia. Given Kazakhstan’s long history of inter-regional animal movement and breed amalgamation, maintaining these genetic reservoirs through targeted breeding strategies, such as lineage-informed registries and monitoring of haplogroup frequencies, will be essential. Additionally, if haplogroup C does persist in low-frequency populations, as suggested by Mukhametzharova et al.’s results, efforts should be made to sample and preserve these rare maternal lineages. In practical terms, conservation actions should include the establishment of lineage-informed breed registries, cryopreservation of germplasm from key maternal lines, and targeted sampling coupled with genomic screening of flocks where rare haplogroups may still persist. These measures will help safeguard genetic diversity and ensure that the adaptive potential of Kazakh fat-tailed sheep is retained for future breeding and management programs.

## 5. Conclusions

Kazakhstani fat-tailed sheep exhibit high haplotype diversity and an unusual balance between haplogroups A and B, reflecting the country’s role as a meeting point for eastern and western maternal lineages. Population analyses demonstrate high within-breed variation, minimal differentiation among breeds, and signatures of demographic expansion, patterns consistent with long-standing livestock mobility and genetic exchange across the Eurasian steppe.

The phylogenetic placement of the Edilbay breed near the most recent common ancestor of domestic sheep and wild *Ovis* may indicate that it retains elements of an ancestral Central Asian lineage, although this should be regarded as a working hypothesis rather than a definitive conclusion. Preserving such breeds could, therefore, help safeguard maternal lineages of potential historical significance.

Together, these findings highlight Kazakhstan as both a reservoir of genetic diversity and a crossroads in sheep domestication history, underscoring the importance of conservation and breeding strategies that maintain within-breed diversity while also protecting rare or potentially ancestral lineages.

## Figures and Tables

**Table 1:** Sampling information of *O. aries* from Kazakhstan.

Breed	Flock	Location	Ns	Farm
Edilbay (ED)	ATR	Atyrau region, WKZ	18	“Suyindik”
	ORL	Birlik village, Zhangaly district, WKZ	20	“Birlik”
	AKZ	Ayakoz town, EKZ	20	“Aigul”
Kazakh fat-tailed coarse-wooled (KFTCW)	KDZ	Kundyzdy village, Abay region, EKZ	20	“Kundyzdy”
	TRZ	Shokpar village, Shu district, Zhambyl region, SKZ	18	“Aksunkar”
Gissar (GISS)	SHK	Darbaza village, Turkestan region, SKZ	19	“Darbaza”

Ns—number of samples; ATR—Atyrau, ORL—Oral, AKZ—Ayakoz, KDZ—Kundyzdy, TRZ—Taraz, SHK—Shymkent. Note: the flock names were employed in the analyses and throughout the text.

**Table 2:** Molecular diversity statistics for three breeds of *O. aries* from Kazakhstan. N—number of samples; Nh—number of haplotypes; S—number of polymorphic sites; m—number of mutations; k—average number of nucleotide differences; Hd—haplotype diversity; π—nucleotide diversity; st.dev.—standard deviations.

Breed	Ns	S	m	k	Nh	Hd ± st.dev.	π ± st.dev.
GISS	58	98	99	23.200	54	0.997 ± 0.004	0.02187 ± 0.00275
KFTCW	38	79	79	21.438	27	0.976 ± 0.014	0.02528 ± 0.00115
ED	19	57	57	18.549	17	0.988 ± 0.021	0.02736 ± 0.00074
Total	115	118	121	22.255	98	0.996 ± 0.002	0.02624 ± 0.00048

**Table 3:** Population dynamics indices of Kazakhstani *O. aries* based on mtDNA. Fu and Li’s *D* and *F* neutrality test statistics (*p* > 0.10); Rag—raggedness index, R^2^—Ramos-Onsins index.

Breed	Fu and Li’s *D*	Fu and Li’s *F*	Rag	R^2^
ED	0.37616	0.41342	0.0024	0.1155
KFTCW	0.44014	0.55362	0.0058	0.1313
GISS	0.42196	0.53936	0.0151	0.1524
Total	−0.11543	−0.11554	0.0012	0.0927

**Table 4:** Analysis of Molecular Variance (AMOVA) based on D-loop sequences in Kazakhstani sheep.

Source of Variation	d.f.	Sum of Squares	Variance Components	Percentage of Variation
Among groups	2	43.814	−0.34252 Va	−3.06
Among populations within groups	3	102.001	1.23505 Vb	11.03
Within populations	109	1122.758	10.30054 Vc	92.03
Total	114	1268.574	11.19307	

d.f.—degrees of freedom; Va—variance among groups (between breeds); Vb—variance among populations within groups (among flocks within each breed); Vc—variance within populations (within each flock).

## Data Availability

All novel sequences obtained in this study were deposited to the GenBank under accession numbers PV963845-PV963959.

## Supplementary Materials

The following supporting information can be downloaded at https://www.mdpi.com/article/10.3390/vetsci12100988/s1, Table S1: Taxon set used in this study for the phylogenetic analysis and haplotype network. Table S2. Tajima’s *D* and Fu’s *Fs* neutrality test indices.

## Abbreviations

The following abbreviations are used in this manuscript:

DNA	Deoxyribonucleic acid
KZ	Kazakhstan
D-loop	Displacement loop (mitochondrial control region)
