# Supplementary material for: Genetic Diversity and Population Structure of Fat-Tailed Coarse-Wooled Sheep Breeds Ovis aries from Kazakhstan
Source: Vet Sci. 2025 Oct 13;12(10):988. doi: 10.3390/vetsci12100988 (PMC12568133; doi:10.3390/vetsci12100988)
Supplement: Supplementary file 1 [file vetsci-12-00988-s001.zip › vetsci-3878826-supplementary.pdf]

Table S1. Taxon set used in the study for the phylogenetic analysis and haplotype network.

Voucher	GenBank Accs. No.	HPG	Taxon (breed)	Country	Source
**Kazakhstani sheep**					
1_TRZ	PV963845	-	*O. aries* (KFTCW)	Kazakhstan	This study
2_TRZ	PV963846	HPGA	*O. aries* (KFTCW)	Kazakhstan	This study
3_TRZ	PV963847	HPGA	*O. aries* (KFTCW)	Kazakhstan	This study
4_TRZ	PV963848	HPGA	*O. aries* (KFTCW)	Kazakhstan	This study
5_TRZ	PV963849	HPGA	*O. aries* (KFTCW)	Kazakhstan	This study
6_TRZ	PV963850	HPGB	*O. aries* (KFTCW)	Kazakhstan	This study
7_TRZ	PV963851	HPGA	*O. aries* (KFTCW)	Kazakhstan	This study
8_TRZ	PV963852	HPGA	*O. aries* (KFTCW)	Kazakhstan	This study
9_TRZ	PV963853	HPGB	*O. aries* (KFTCW)	Kazakhstan	This study
10_TRZ	PV963854	HPGA	*O. aries* (KFTCW)	Kazakhstan	This study
11_TRZ	PV963855	HPGA	*O. aries* (KFTCW)	Kazakhstan	This study
12_TRZ	PV963856	HPGB	*O. aries* (KFTCW)	Kazakhstan	This study
13_TRZ	PV963857	HPGB	*O. aries* (KFTCW)	Kazakhstan	This study
14_TRZ	PV963858	HPGB	*O. aries* (KFTCW)	Kazakhstan	This study
15_TRZ	PV963859	HPGA	*O. aries* (KFTCW)	Kazakhstan	This study
16_TRZ	PV963860	HPGA	*O. aries* (KFTCW)	Kazakhstan	This study
17_TRZ	PV963861	HPGA	*O. aries* (KFTCW)	Kazakhstan	This study
18_TRZ	PV963862	HPGA	*O. aries* (KFTCW)	Kazakhstan	This study
1_SHK	PV963863	HPGB	*O. aries* (Gissar)	Kazakhstan	This study
2_SHK	PV963864	-	*O. aries* (Gissar)	Kazakhstan	This study
3_SHK	PV963865	HPGB	*O. aries* (Gissar)	Kazakhstan	This study
4_SHK	PV963866	HPGA	*O. aries* (Gissar)	Kazakhstan	This study
5_SHK	PV963867	HPGA	*O. aries* (Gissar)	Kazakhstan	This study
6_SHK	PV963868	HPGA	*O. aries* (Gissar)	Kazakhstan	This study
7_SHK	PV963869	HPGA	*O. aries* (Gissar)	Kazakhstan	This study
8_SHK	PV963870	HPGB	*O. aries* (Gissar)	Kazakhstan	This study
9_SHK	PV963871	HPGB	*O. aries* (Gissar)	Kazakhstan	This study
10_SHK	PV963872	HPGA	*O. aries* (Gissar)	Kazakhstan	This study
11_SHK	PV963873	HPGA	*O. aries* (Gissar)	Kazakhstan	This study
12_SHK	PV963874	HPGB	*O. aries* (Gissar)	Kazakhstan	This study
13_SHK	PV963875	HPGA	*O. aries* (Gissar)	Kazakhstan	This study
14_SHK	PV963876	HPGA	*O. aries* (Gissar)	Kazakhstan	This study
15_SHK	PV963877	HPGA	*O. aries* (Gissar)	Kazakhstan	This study
16_SHK	PV963878	HPGA	*O. aries* (Gissar)	Kazakhstan	This study
17_SHK	PV963879	HPGA	*O. aries* (Gissar)	Kazakhstan	This study
18_SHK	PV963880	HPGA	*O. aries* (Gissar)	Kazakhstan	This study
19_SHK	PV963881	HPGA	*O. aries* (Gissar)	Kazakhstan	This study
1_ORL	PV963882	HPGA	*O. aries* (Edilbay)	Kazakhstan	This study
2_ORL	PV963883	HPGB	*O. aries* (Edilbay)	Kazakhstan	This study
3_ORL	PV963884	HPGA	*O. aries* (Edilbay)	Kazakhstan	This study
4_ORL	PV963885	HPGA	*O. aries* (Edilbay)	Kazakhstan	This study
5_ORL	PV963886	HPGA	*O. aries* (Edilbay)	Kazakhstan	This study
6_ORL	PV963887	HPGB	*O. aries* (Edilbay)	Kazakhstan	This study
7_ORL	PV963888	HPGB	*O. aries* (Edilbay)	Kazakhstan	This study
8_ORL	PV963889	HPGA	*O. aries* (Edilbay)	Kazakhstan	This study
9_ORL	PV963890	HPGB	*O. aries* (Edilbay)	Kazakhstan	This study
10_ORL	PV963891	HPGB	*O. aries* (Edilbay)	Kazakhstan	This study
11_ORL	PV963892	HPGA	*O. aries* (Edilbay)	Kazakhstan	This study
12_ORL	PV963893	HPGA	*O. aries* (Edilbay)	Kazakhstan	This study
13_ORL	PV963894	HPGA	*O. aries* (Edilbay)	Kazakhstan	This study
14_ORL	PV963895	HPGA	*O. aries* (Edilbay)	Kazakhstan	This study
15_ORL	PV963896	HPGB	*O. aries* (Edilbay)	Kazakhstan	This study
16_ORL	PV963897	HPGB	*O. aries* (Edilbay)	Kazakhstan	This study
17_ORL	PV963898	HPGB	*O. aries* (Edilbay)	Kazakhstan	This study
18_ORL	PV963899	HPGA	*O. aries* (Edilbay)	Kazakhstan	This study

19_ORL	PV963900	HPGA	*O. aries* (Edilbay)	Kazakhstan	This study
20_ORL	PV963901	HPGB	*O. aries* (Edilbay)	Kazakhstan	This study
1_ATR	PV963902	HPGB	*O. aries* (Edilbay)	Kazakhstan	This study
2_ATR	PV963903	HPGA	*O. aries* (Edilbay)	Kazakhstan	This study
3_ATR	PV963904	HPGB	*O. aries* (Edilbay)	Kazakhstan	This study
4_ATR	PV963905	HPGA	*O. aries* (Edilbay)	Kazakhstan	This study
5_ATR	PV963906	HPGA	*O. aries* (Edilbay)	Kazakhstan	This study
6_ATR	PV963907	HPGB	*O. aries* (Edilbay)	Kazakhstan	This study
7_ATR	PV963908	HPGB	*O. aries* (Edilbay)	Kazakhstan	This study
8_ATR	PV963909	HPGA	*O. aries* (Edilbay)	Kazakhstan	This study
9_ATR	PV963910	HPGB	*O. aries* (Edilbay)	Kazakhstan	This study
10_ATR	PV963911	HPGA	*O. aries* (Edilbay)	Kazakhstan	This study
11_ATR	PV963912	HPGB	*O. aries* (Edilbay)	Kazakhstan	This study
12_ATR	PV963913	HPGA	*O. aries* (Edilbay)	Kazakhstan	This study
13_ATR	PV963914	HPGA	*O. aries* (Edilbay)	Kazakhstan	This study
14_ATR	PV963915	HPGA	*O. aries* (Edilbay)	Kazakhstan	This study
15_ATR	PV963916	-	*O. aries* (Edilbay)	Kazakhstan	This study
16_ATR	PV963917	HPGB	*O. aries* (Edilbay)	Kazakhstan	This study
17_ATR	PV963918	HPGB	*O. aries* (Edilbay)	Kazakhstan	This study
18_ATR	PV963919	HPGB	*O. aries* (Edilbay)	Kazakhstan	This study
1_AKZ	PV963920	HPGA	*O. aries* (Edilbay)	Kazakhstan	This study
2_AKZ	PV963921	HPGA	*O. aries* (Edilbay)	Kazakhstan	This study
3_AKZ	PV963922	HPGB	*O. aries* (Edilbay)	Kazakhstan	This study
4_AKZ	PV963923	HPGA	*O. aries* (Edilbay)	Kazakhstan	This study
5_AKZ	PV963924	-	*O. aries* (Edilbay)	Kazakhstan	This study
6_AKZ	PV963925	-	*O. aries* (Edilbay)	Kazakhstan	This study
7_AKZ	PV963926	-	*O. aries* (Edilbay)	Kazakhstan	This study
8_AKZ	PV963927	HPGA	*O. aries* (Edilbay)	Kazakhstan	This study
9_AKZ	PV963928	HPGA	*O. aries* (Edilbay)	Kazakhstan	This study
10_AKZ	PV963929	HPGA	*O. aries* (Edilbay)	Kazakhstan	This study
11_AKZ	PV963930	-	*O. aries* (Edilbay)	Kazakhstan	This study
12_AKZ	PV963931	-	*O. aries* (Edilbay)	Kazakhstan	This study
13_AKZ	PV963932	HPGB	*O. aries* (Edilbay)	Kazakhstan	This study
14_AKZ	PV963933	HPGB	*O. aries* (Edilbay)	Kazakhstan	This study
15_AKZ	PV963934	HPGA	*O. aries* (Edilbay)	Kazakhstan	This study
16_AKZ	PV963935	HPGA	*O. aries* (Edilbay)	Kazakhstan	This study
17_AKZ	PV963936	HPGA	*O. aries* (Edilbay)	Kazakhstan	This study
18_AKZ	PV963937	-	*O. aries* (Edilbay)	Kazakhstan	This study
19_AKZ	PV963938	HPGA	*O. aries* (Edilbay)	Kazakhstan	This study
20_AKZ	PV963939	HPGA	*O. aries* (Edilbay)	Kazakhstan	This study
1_KDZ	PV963940	HPGB	*O. aries* (KFTCW)	Kazakhstan	This study
2_KDZ	PV963941	HPGB	*O. aries* (KFTCW)	Kazakhstan	This study
3_KDZ	PV963942	HPGB	*O. aries* (KFTCW)	Kazakhstan	This study
4_KDZ	PV963943	-	*O. aries* (KFTCW)	Kazakhstan	This study
5_KDZ	PV963944	HPGB	*O. aries* (KFTCW)	Kazakhstan	This study
6_KDZ	PV963945	-	*O. aries* (KFTCW)	Kazakhstan	This study
7_KDZ	PV963946	HPGA	*O. aries* (KFTCW)	Kazakhstan	This study
8_KDZ	PV963947	HPGB	*O. aries* (KFTCW)	Kazakhstan	This study
9_KDZ	PV963948	HPGB	*O. aries* (KFTCW)	Kazakhstan	This study
10_KDZ	PV963949	HPGA	*O. aries* (KFTCW)	Kazakhstan	This study
11_KDZ	PV963950	HPGA	*O. aries* (KFTCW)	Kazakhstan	This study
12_KDZ	PV963951	HPGB	*O. aries* (KFTCW)	Kazakhstan	This study
13_KDZ	PV963952	HPGB	*O. aries* (KFTCW)	Kazakhstan	This study
14_KDZ	PV963953	HPGB	*O. aries* (KFTCW)	Kazakhstan	This study
15_KDZ	PV963954	HPGB	*O. aries* (KFTCW)	Kazakhstan	This study
16_KDZ	PV963955	HPGB	*O. aries* (KFTCW)	Kazakhstan	This study
17_KDZ	PV963956	HPGB	*O. aries* (KFTCW)	Kazakhstan	This study
18_KDZ	PV963957	-	*O. aries* (KFTCW)	Kazakhstan	This study
19_KDZ	PV963958	HPGB	*O. aries* (KFTCW)	Kazakhstan	This study

20_KDZ	PV963959	HPGB	*O. aries* (KFTCW)	Kazakhstan	This study
**External GenBank sequences**					
	OQ440008	HPGA	*O. aries* (Gissar)		Koshkina et al., 2023 [32]
	OQ440009	HPGB	*O. aries* (Gissar)		Koshkina et al., 2023 [32]
	OR459640	HPGB	*O. aries* (Dagestan)		Koshkina et al., 2023 [32]
	OR459703	HPGB	*O. aries* (Kalmyk)		Koshkina et al., 2023 [32]
	OR457704	HPGB	*O. aries* (Kalmyk))		Koshkina et al., 2023 [32]
	OR459705	HPGB	*O. aries* (Kalmyk)		Koshkina et al., 2023 [32]
	OR459706		*O. aries* (Kalmyk)		Koshkina et al., 2023 [32]
	OR459718	HPGB	*O. aries* (Edilbay)		Koshkina et al., 2023 [32]
	OR459719	HPGA	*O. aries* (Edilbay)		Koshkina et al., 2023 [32]
	OR459720	HPGB	*O. aries* (Edilbay)		Koshkina et al., 2023 [32]
	OR459721	HPGB	*O. aries* (Edilbay)		Koshkina et al., 2023 [32]
	OR459722	HPGB	*O. aries* (Edilbay)		Koshkina et al., 2023 [32]
	OR459723	HPGB	*O. aries* (Edilbay)		Koshkina et al., 2023 [32]
	OR459724	HPGB	*O. aries* (Edilbay)		Koshkina et al., 2023 [32]
cl122	HM236174	HPGA	*O. aries* (Merino)	Australia	Meadows et al., 2011 [6]
r359	HM236175	HPGA	*O. aries* (Romney)	Australia	Meadows et al., 2011 [6]
kk1	HM236176	HPGB	*O. aries* (Karakas)	Turkey	Meadows et al., 2011 [6]
kk2	HM236177	HPGB	*O. aries* (Karakas)	Turkey	Meadows et al., 2011 [6]
	HM236178	HPGC	*O. aries* (Karakas)	Turkey	Meadows et al., 2011 [6]
	HM236179	HPGC	*O. aries* (Morkaraman)	Turkey	Meadows et al., 2011 [6]
mk3	HM236180	HPGD	*O. aries* (Morkaraman)	Turkey	Meadows et al., 2011 [6]
mk9	HM236181	HPGD	*O. aries* (Morkaraman)	Turkey	Meadows et al., 2011 [6]
aw25	HM236182	HPGE	*O. aries* (Awassi)	Israel	Meadows et al., 2011 [6]
tj6	HM236183	HPGE	*O. aries* (Tuj)	Turkey	Meadows et al., 2011 [6]
	KF312238		*O. o. ophion* (= *O. gmelini ophion*, Cyprus mouflon)	Cyprus	Sana et al., 2015 [34]
	KF938360		*O. orientalis* (Asian mouflon)		Lv et al., 2015 [33]
	KF938361		*O. vignei* (Urial)		Lv et al., 2015 [33]
	HM236188		*O. ammon* (Argali)	Kazakhstan	Meadows et al., 2011[6]

Source citation given to Table S1 is consistent with the manuscript`s References list. (-) indicating the transitional haplotypes, which not belongs to any of the known haplogroups.

Table S2. Tajima`s *D* and Fu`s *Fs* neutrality test indices.

**Neutrality test**	**ED**	**KFTCW**	**GISS**
Tajima`s *D* (*p* - value)	0.00793 (p = 0.59300)	0.64043 (p = 0.78200)	0.36876 (p = 0.69600)
Fu`s *Fs* (*p* - value)	−24.10886 (p = 0.00000)	−19.66488 (p = 0.00000)	−6.10774 (p = 0.01200)
